# Functional Dissection of Sugar Signals Affecting Gene Expression in *Arabidopsis thaliana*

**DOI:** 10.1371/journal.pone.0100312

**Published:** 2014-06-20

**Authors:** Sabine Kunz, Edouard Pesquet, Leszek A. Kleczkowski

**Affiliations:** Department of Plant Physiology, Umeå Plant Science Centre, Umeå University, Umeå, Sweden; National Taiwan University, Taiwan

## Abstract

**Background:**

Sugars modulate expression of hundreds of genes in plants. Previous studies on sugar signaling, using intact plants or plant tissues, were hampered by tissue heterogeneity, uneven sugar transport and/or inter-conversions of the applied sugars. This, in turn, could obscure the identity of a specific sugar that acts as a signal affecting expression of given gene in a given tissue or cell-type.

**Methodology/Principal Findings:**

To bypass those biases, we have developed a novel biological system, based on stem-cell-like *Arabidopsis* suspension culture. The cells were grown in a hormone-free medium and were sustained on xylose as the only carbon source. Using functional genomics we have identified 290 sugar responsive genes, responding rapidly (within 1 h) and specifically to low concentration (1 mM) of glucose, fructose and/or sucrose. For selected genes, the true nature of the signaling sugar molecules and sites of sugar perception were further clarified using non-metabolizable sugar analogues. Using both transgenic and wild-type *A. thaliana* seedlings, it was shown that the expression of selected sugar-responsive genes was not restricted to a specific tissue or cell type and responded to photoperiod-related changes in sugar availability. This suggested that sugar-responsiveness of genes identified in the cell culture system was not biased toward heterotrophic background and resembled that in whole plants.

**Conclusions:**

Altogether, our research strategy, using a combination of cell culture and whole plants, has provided an unequivocal evidence for the identity of sugar-responsive genes and the identity of the sugar signaling molecules, independently from their inter-conversions or use for energy metabolism.

## Introduction

Daily changes in the environment of green plants result in constant fluctuation in the contents of endogenous sugars, which – besides being the initial carbon backbone for biological molecules - represent the main energy source in both the autotrophic and heterotrophic tissues. Primary sugars in plants include glucose (Glc), fructose (Fru) and sucrose (Suc), which derive from photosynthesis and storage reserves. Their fine-tuned distribution from the carbon-fixing and reserve cells to the rest of the organism is essential for the optimal plant growth and development [1]. Besides their role as energy resource, these sugars also act as signaling molecules, modulating the expression of the so called “sugar-responsive” genes. In plants, there are hundreds of such genes, involved both in the cellular adjustment to nutrient availability and controling resource distribution between tissues and organs [2]–[4]. Studies on sugar-responsive genes used a variety of biological experimental systems, ranging from mesophyll protoplasts [5] and cell culture [6], to exposing the seedlings to a given sugar-containing media [7], [8], feeding detached leaves with a given sugar ([9], [10] and studying gene expression in whole plants under conditions of physiological depletion of endogenous sugars (e.g. end of the night) [4] or in mutants with impaired endogenous sugar levels [11]. Based on some of those studies, independent signaling pathways have been postulated for Glc, Fru and Suc [5], [8], [10].

When feeding specific sugars to intact plants or excised leaves, it is frequently not clear how fast and evenly the taken up carbohydrates are transported within the tissue and whether a given sugar is converted into related sugar species, which in turn might act as a distinct signal inducing changes in gene expression ([12]. Cells lying on the exterior of the treated plant face a higher sugar concentration than cells on the inside of the plant, leading to gradients in gene expression from cell layer to cell layer. Also, a sample derived from a whole seedling or leaf rosette always consists of cells in different developmental stages, possessing a variety of specialized functions and distinct metabolism. This complicates the distinction between direct and indirect consequences of sugar treatment. A homogenous response would be expected with cell culture, but those cells usually require an addition of both sugars and hormones to sustain their division and growth, which could affect sugar perception and metabolism. Studies using a mesophyll protoplast transient expression system [5] partly overcome this difficulty; however, these photosynthetic cells - devoid of cell walls and composed of multiple cell types (e.g. spongy and palisade mesophyll) - represent a system which cannot be reliably extended to whole plants when studying global expression.

The choice of proper experimental system to study signaling, whether by sugars or other compounds, represents a serious challenge in all fields of biology. In the medical field, stem-cell research has allowed for pioneering studies by using a homogenous cell population (without interference from other cell types and/or morphogenic compounds), which could be induced to differentiate into specialized cell-types, e.g. neuron [13] or vascular precursor ([14]. Stem-cell studies in plants have similarly enabled breakthroughs in understanding key compounds controlling the cell status from active proliferation to differentiation [15], [16]. In the present study, in order to bypass the previously-mentioned biases inherent to studies on sugar signaling, we have developed a novel system, based on the use of a stem-cell-like suspension culture of *Arabidopsis thaliana* which could be grown without addition of any hormones [17]. Using functional genomics in this biological system, we have identified and characterized genes that rapidly respond to low sugar concentration and have unravelled true nature of the signaling sugar species and their perception sites. The sugar responsiveness of selected genes was then verified *in planta* using *Arabidopsis* seedlings. The findings, combining the use of habituated cell culture and whole plants, shed new light on sugar signaling in plants.

## Materials and Methods

### Cell culture maintenance and growth characteristics

Habituated stem-cell-like suspension culture used in this study was derived from roots of *A. thaliana* [17]. The cells were grown on an orbital shaker in the dark in full-strength MS liquid media. Commercially available MS salts with vitamins (Duchefa, Haarlem, Netherlands) were prepared and autoclaved prior to the addition of the carbon source. Filter sterilized stock solutions of Suc and the pentose D-(+)-Xyl (Sigma-Aldrich) were added to the liquid media to reach a final concentration of 87 mM. The cells were subcultured every 7 d through harvesting cells by centrifugation for 2 min at 200xg, measuring the packed cell volume (PCV), washing the cells with fresh media and subdividing the culture in additional fresh media with a ratio 1∶2 (Xyl-grown) or 1∶8 (Suc-grown). To estimate the effect of the C-source on the growth, *A. thaliana* cells, conditioned for growth on either Suc- or Xyl-containing MS media, were subcultured into the respective Suc- or Xyl-containing media and incubated for 7 days in the dark. Every day, samples were taken and the PCV of each sample was estimated after centrifugation for 2 min at 200xg. Subsequently, the media was removed through vacuum filtration and the cells were washed twice using full strength MS-media without C-source. The dry cell pellet was immediately frozen in liquid N_2_ and stored at −80°C for further analysis.

### Cell culture induction conditions

All cell culture induction assays to estimate the effect of exogenously supplied sugar or sugar analogues were done using 7-d-old Xyl-grown cells, which were not sub-cultured or transferred to fresh media prior to the experiment. Homogeneous cell solutions were equally divided onto conical flasks prior to the treatment. Induction of cells with 0, 1 and 5 mM filter-sterilized Suc was conducted for 0, 5, 15 and 30 min as well as 1 and 3 h on an orbital shaker in the dark. Samples for the microarray study were collected after a 1 h incubation of Xyl-grown cells with 1 mM filter sterilized Xyl, Suc, Glc and Fru, respectively. The effect of non-metabolizable sugar analogues on gene expression was tested through the application of 1 mM filter-sterilized Xyl, Suc, D-Glc, Fru, turanose, palatinose, 2-deoxyglucose, L-Glc and mannitol to 7-d-old Xyl-grown cells, respectively. After treatment, the culture media was removed through vacuum filtration. The cell pellet was washed 2 times with full strength MS-media without sugar and, after drying, immediately snap-frozen and ground in liquid N_2_. The cell powder was stored at -80°C until further use.

### Plant material

Seedlings of *Arabidopsis thaliana* col-0 wt, *pgm1* mutant and *promoter::GUS* lines for the genes *bZIP63*, *MRS2.11*, *URH1* (Col-0 background) and *TOR* (Wassilijewska background) were grown *in vitro* on vertical plates containing a half-strength MS agar without sugar supplement. The harvest was carried out at the developmental stage of the appearance of the second pair of real leaves, which occurred after 13 days of growth in a long day growth chamber (16 h light, 8 h dark; 150 µmol m^−2^ s^−2^). Seedlings used for RNA extraction were harvested at the end of the light period and at the end of a 6 h extended night, by separating roots from shoots and immediately snap-freezing the material in liquid N_2_. Seedlings used for GUS-analysis were harvested at the end of the light period and directly transferred into GUS containing staining solution.

### RNA extraction and cDNA synthesis

Total RNA from samples after Suc-treatments and sample preparation for the microarray was isolated by applying TRI Reagent (Sigma-Aldrich) and chloroform to frozen and ground plant material. Phase separation was reached by centrifugation at full speed for 15 min. The aqueous fraction was mixed with half a volume isopropanol and the nucleic acids were pelleted by a 10 min centrifugation at full speed. The pellet was washed 2 times with 70% ethanol, air-dried and subsequently taken up in sterile water. The total RNA samples were DNase-treated using RQ1 RNase-Free DNase (Promega Biotech AB) and RNA was re-extracted as described above. Total RNA from samples after sugar analogue treatments as well as seedling material was isolated by using the E.Z.N.A Plant RNA kit from OMEGA BioTek (R6827-01). After elution of the RNA from the column, possible DNA contaminations were removed through a DNase treatment using the Ambion DNA-*free* DNase treatment and removal kit (AM1906). cDNA was generated using SuperScript II Reverse Transcriptase (Invitrogen) and Oligo(dT)_18_ primers (Fermentas Life Science) following the manufacturers protocol. 1 µg total RNA was used in each reaction.

### Gene expression analysis

Quantitative Real-time PCR was conducted using Roche LightCycler 480 Real Time PCR system and Roche LightCycler 480 SYBR Green I Master following the manufacturer's protocol. Sequences of RT-PCR primers for all tested genes are listed in **Table S1**. *TUB5* was used as endogenous reference gene in samples derived from *A. thaliana* cell culture after analysis of the microarray data showed no changes in the expression in response to sugar treatment. In samples derived from *A. thaliana* seedlings, *PP2A* was used as endogenous reference gene. The shown data represent means±SD of the normalized expression in relation to the expression of the respective reference gene. To assess the statistical significance of differences in gene expression between the various treatments, a Student's *t*-test (two-tailed distribution of two samples with unequal variance) was applied.

The analysis of the transcriptome, using the Affymetrix GeneChip ATH1 genome array on 3 biological replicates per treatment was done by the SciLife Lab (Uppsala, Sweden). The resulting signal values were transformed into expression data and normalized using the RMA normalization algorithm. Subsequently, the ratios of the gene expression of each gene in Suc-, Glc-, Fru- to Xyl-treated cells were calculated, respectively. Finally p-values were calculated using Student's *t*-test. Genes showing p-values <0.002 were selected as differentially expressed and sorted into non-redundant lists with genes responsive to Suc/Glc/Fru, Suc/Fru, Suc/Glc, Fru/Glc, Suc, Glc or Fru, respectively. Hierarchical clustering based on the Pearson correlation of the expression profile of the selected genes was performed using the Hierarchical Clustering Explorer v.3.5 software. The list with selected differentially expressed genes was cross-referenced with lists of sugar-responsive genes deriving from other global studies listed in Excel file **(Dataset S1)**. In addition the expression of the selected differentially expressed genes was examined throughout expression datasets generated from different plants organs (all available material, except data generated from cell culture) and sugar treatments (AT-0006; 0014; 0015; 0056; 0133; 0199; 0209; 0281; 0639; 0650.) using the Genevestigator V3 software [18]. With this software, hierarchical clustering based on the Pearson correlation of the expression profiles of the selected genes was conducted on the extracted data.

Microarray data have been deposited in NCBI's Gene Expression Omnibus and are accessible through GEO Series accession number GSE46510: (http://www.ncbi.nlm.nih.gov/geo/query/acc.cgi?acc=GSE46510).

### Carbohydrate analyses

Frozen cell material was ground in liquid N_2_. The soluble sugars sucrose, glucose and fructose were analyzed in ethanol-water extracts. Sugars were extracted in 80% ethanol containing 4 mM Hepes, pH 7.5, at 80°C for 15 min. After 10 min centrifugation at full speed the supernatant was collected, stored on ice and the extraction was repeated. Subsequently the extraction was repeated with 50% ethanol containing 4 mM Hepes, pH 7.5. All supernatants were combined and subsequently analyzed for sugar contents using a NADP-coupled enzyme assay. The remaining pellets were resuspended in 200 mM citrate (pH 4.6), incubated for 20 min at 95°C, cooled down and digested over night at 55°C by amyloglucosidase (Roche). After a subsequent centrifugation at full speed for 10 min, the supernatant was transferred and analyzed for sugar contents using a NADP-coupled enzyme assay. In brief, first all glucose was consumed by the enzymes glucose-6-phosphate-dehydrogenase (Roche) and hexokinase (Roche) in the reaction buffer (100 mM imidazol, 1.5 mM MgCl2; pH 6.9, 0.5 mM NADP; 1.25 mM ATP) and the OD_344_ was monitored until a plateau. Subsequently all fructose was consumed by the addition of the enzyme phosphoglucoisomerase (Roche), which could be monitored by the increase of the OD_344_. After reaching a plateau, finally all sucrose was consumed through the addition of the enzyme invertase (Roche). OD_344_ was monitored until a plateau was reached. Based on the differences between the OD_344_ at the plateau-stages for each sugar, the sugar concentration for glucose, fructose and sucrose were calculated in µmol hexose/gFW.

### GUS staining

13-day-old whole seedlings were transferred into GUS-staining solution (1 mM X-Glc (Fermentas/Thermo Scientific); 50 mM NaHPO_4_, pH 7.0; 1% v/v TritonX-100). The samples were incubated overnight at 37 °C. Afterwards the samples were washed twice for 2 min in 50% ethanol, twice for 2 min in 90% ethanol and finally rinsed and stored in sterile MilliQ-water. The samples were analyzed by taking pictures using a Canon EOS 650D.

## Results and Discussion

### Novel cell system to identify and investigate sugar-responsive genes

In order to identify sugar-responsive genes independently from **(i)** the developmental and cellular heterogeneity of plant tissues, **(ii)** the differential accessibility to sugars and **(iii)** the presence of the studied sugar as energy source, a simplified biological system was adapted using habituated stem-cell-like suspension cultures from *A. thaliana* [17]. The system facilitates a homogeneous and direct response towards externally applied primary sugars, while excluding tissue- or development-specific signals that could affect the sugar-dependent gene expression. Contrary to most cell cultures, the habituated cells have the capacity to grow and divide in a hormone-free medium and possess the ability to differentiate if provided with specific hormones [17]. This makes them an ideal model to study cellular response to a specific factor.

**Figure 1:**
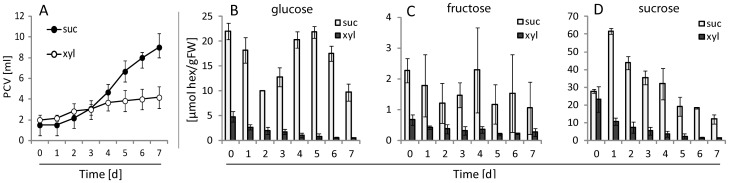
Effects of Xyl and Suc, as C-sources in the growth media, on packed cell volume (PCV) and intracellular soluble sugar concentration in *A. thaliana* cell culture. (A) Cells-grown in 1x MS medium supplemented with Xyl or Suc. (B-D) Internal concentrations of Glc, Fru and Suc after 7 d of growth on Xyl or Suc media. Error bars represent the standard deviation calculated from 3 biological repeats.

To effectively decipher between sugars used as energy source or as signals, cell suspensions were screened for growth on Murashige and Skoog (MS) media supplemented with 87 mM of different sugars. The pentose xylose (Xyl) was identified as a usable carbon source, along with Suc, Glc, Fru, trehalose, raffinose, maltose and lactose, providing sufficient energy in the culture media for sustained growth over a 7 day growth period **(Fig. S1A)**. In plants, Xyl can be converted to Glc via the oxidative pentose-phosphate pathway [19] and the Xyl-based growth was earlier reported both for cultured plant cells [20] and for *Arabidopsis* seedlings kept on Xyl-supplemented growth media [21]. In our system, the growth of cells conditioned to grow on Xyl-containing media was about 2.5-fold slower than those conditioned to grow on Suc **(** **Fig. 1A** **)**, but cell length and general appearance of the cells under those two conditions were similar **(Fig. S1B,C)**. Along with the decreased growth, the Xyl-grown cells had reduced contents of internal soluble sugars, with Glc, Fru and Suc levels decreased by 20-, 4- and 9-fold, respectively, when compared to Suc-grown cells **(** **Fig. 1B-D** **)**. Thus, under long-term low-carbon conditions, the cells adapted by establishing a decreased growth rate, similar to a whole plant response when kept under prolonged low carbon regime [3].

**Figure 2:**
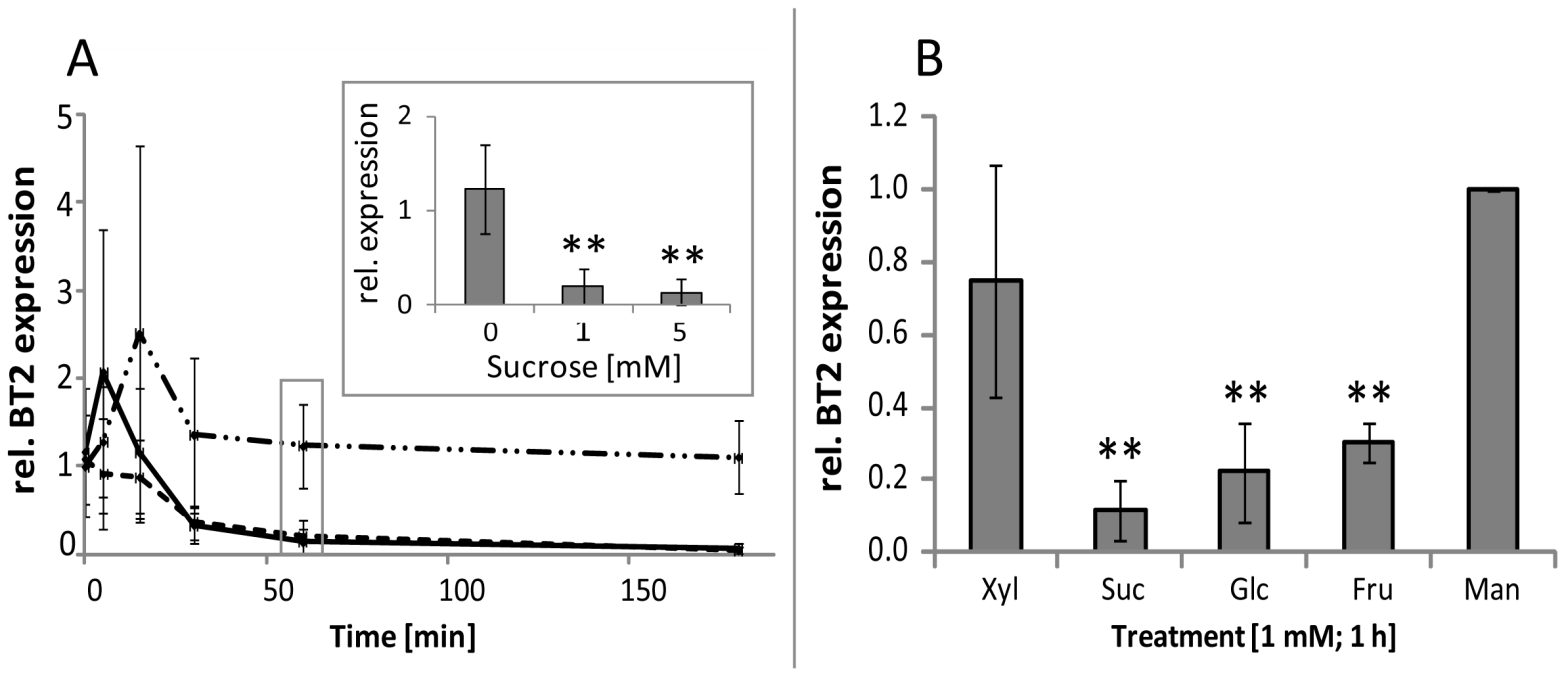
A rapid response of *BT2* expression to low doses of sugars in 7-d-old Xyl-grown *A. thaliana* cell culture. **(A)** *BT2* expression in the cell culture supplemented with 0 (dash-dot line), 1 mM (straight) and 5 mM (dashed) Suc for 0, 5, 15, 30 min and 1 and 3 h (n = 3). The box represents data for 1 h treatment with Suc. **(B)** Effects of 1 mM Suc, Glc Fru and Xyl on *BT2* expression. Mannitol (Man) at 1 mM was a control. Significance: *t*-test; *α = 0.05, **α = 0.01, n = 7.

Deciphering the role of a sugar as a signaling molecule from its role as energy-source is one of the main problems hampering studies on sugar-mediated regulation of gene expression. Another difficulty is a frequently observed rapid conversion of an externally supplied sugar, especially Suc, to other sugars [12], [22]. We have addressed those difficulties by using the Xyl-grown cells, which were exposed to low or very low sugar concentrations (1 and 5 mM) for a short time (up to 3 h). The results suggested that treatment with as little as 1 mM Suc for 1 h did not increase the specific sugar pools **(Fig. S2)**. Those conditions were also likely to limit the use of sugars as energy source. Next, we assessed the impact of externally added sugars on expression of *BT2* (encoding a BTB- and TAZ-domain containing protein), whose expression was reported to be regulated by sugar and diurnal rhythms in intact plants, with maximum expression in the dark or in carbon-starved seedlings [7], [23]. Using qPCR analysis, *BT2* expression in Xyl-adapted cells was sensitive to exogenous Suc application **(** **Fig. 2A** **)**, with Suc concentration as low as 1 mM being sufficient to significantly decrease *BT2* expression within 1 h **(** **Fig. 2A**, box). A similar down-regulation was also observed after 1 h treatments with either 1 mM Glc or Fru **(** **Fig. 2B** **)**, suggesting that *BT2* responds both to Suc and hexoses, and that growth on Xyl does not impair *BT2* responses to primary sugars. The rapid response of *BT2* expression to a very low sugar concentration prompted us to seek other genes which may rapidly respond to the same sugars under the conditions established for the cell culture.

### Functional genomics identification of genes rapidly regulated by low sugar concentrations

**Figure 3:**
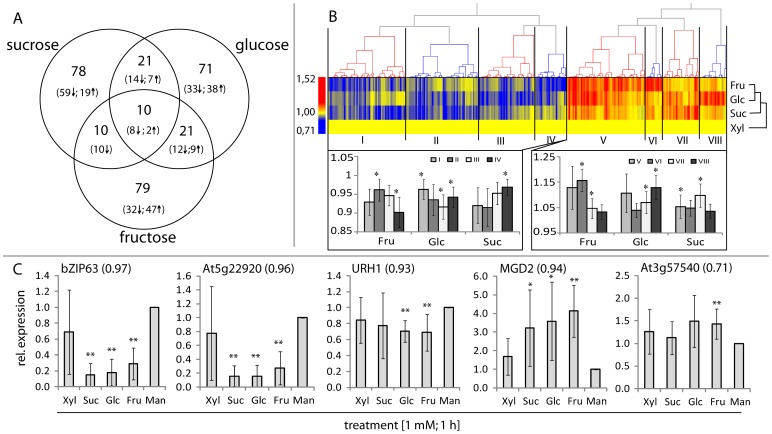
Suc, Glc and Fru induce sugar species-specific changes in global gene expression in *A. thaliana* cell culture. **(A)** Treatment of 7-d-old Xyl-grown cell culture for 1 h with 1 mM Suc, Glc and Fru induced differential expression of 290 genes, which were divided into 7 subgroups, representing their responsiveness to a specific set of sugars. **(B)** Hierarchical clustering of the expression profiles based on Pearson correlation of the 290 genes resulted in 8 clusters with 4 clusters each containing up- or down-regulated genes. The single clusters represented expression profiles responsive to a specific set of sugar species. Significance: *t*-test; * α = 0.01; n = 13 to 59, depending on the cluster. **(C)** qPCR expression analysis of selected genes in response to a specific sugar (at 1 mM for 1 h). Mannitol (Man) at 1 mM was a control. The numbers in parentheses indicate the correlation between the gene expression profiles measured by the microarray and qPCR analysis. Significance: *t*-test; *α = 0.05, **α = 0.01, n = 8. See also **Fig. S7** for examples of other genes.

To identify genes rapidly responding to low concentrations of specific sugars, expression analyses using the Affymetrix ATH1 microarrays were performed on Xyl-based cell-culture samples treated for 1 h with 1 mM of either Glc, Fru, Suc or Xyl in 3 independent replicates for each treatment. Gene expression was calculated as the ratio of the expression value between the treatment with a distinct sugar and the values from Xyl-treated control samples. Xyl was used as a control, rather than e.g. mannitol, since it is penetrable and serves as energy source **(** **Fig. 1** **)**, similar to Glc, Fru and Suc. Selection of significantly expressed genes was performed using stringent criteria (RMA normalization and the threshold p-values <0.002) which identified a total of 290 sugar responsive genes **(Dataset S1)** with expression ratios ranging between 1.52 and 0.71. The genes could be divided into 7 sub-groups, corresponding to those responsive to Suc/Fru/Glc, Fru/Glc, Suc/Glc, Suc/Fru, Suc, Glc, and Fru, respectively **(** **Fig. 3A** **)**. Hierarchical clustering, based on complete Pearson correlation of the expression profiles of the identified genes, revealed distinct clusters exhibiting both a specific direction of expression patterns and responsiveness exclusively to distinct sugar molecules **(** **Fig. 3B** **)**. Out of the 290 sugar-responsive genes, 168 (58%) were down-regulated and 122 (42%) – up-regulated **(** **Fig. 3A** **)**. A similar bias toward down-regulation was frequently observed in studies on auxin- and other hormone-responsive genes (e.g. in [24]). From the sensing and signaling perspective, suppression of gene expression may represent a faster response mechanism than up-regulation of the expression.

The comparison of the 290 candidate genes with three publically available datasets focusing on the transcriptional response to **(i)** sugars and circadian regulation during the diurnal cycle [25], **(ii)** changes in endogenous carbon status [4], and **(iii)** the supplement of 15 mM Suc to C-starved seedlings [7] revealed a substantial overlap of 20% only with genes identified upon Suc-supplement **(Dataset S1)**. The overlapping genes were members of all 7 sub-groups **(Fig. S3)**, suggesting that the *in planta* response to Suc-treatment might have been partly induced by its hydrolytic products Glc and Fru. Similar to our microarray data, the majority of those overlapping genes (47 out of 60) were down-regulated. Further analyses of publically available datasets on intact plants treated with Suc or Glc or exposed to extended dark conditions again revealed some overlap for each of the dataset with the candidate genes identified in the present study **(Fig. S4)**. About 50% of the 290 genes had a 2-fold change in expression in at least one of the conditions analyzed. Expression profiles of the identified genes were distributed in a plethora of tissues **(Fig. S5)**, with no apparent bias toward any tissue. Thus, the expression of sugar responsive genes identified in the present study using an initially root-derived heterotrophic cell culture was not restricted to roots, heterotrophic background, but encompassed both non-photosynthetic and photosynthetic tissues.

Among the 290 candidate genes selected by microarrays, 218 coded for proteins with known function **(Fig. S6)**. Several of the identified sugar-responsive genes, e.g. those involved in endomembrane trafficking, cell wall synthesis, protein degradation, microtubule-associated, etc., were related to the cell division process. Signaling-related sugar-responsive genes included *TOR*, coding for a key integrator of energy status in eukaryotes [26], [27] and *bZIP63*, coding for a transcription factor integrating Glc and ABA signals in plants ([28]. TOR, among other functions, regulates Glc-dependent repression of *BT2* [27], a gene also found as sugar-responsive in our study **(** **Fig. 2** **)**.

Sugar responsiveness of selected genes identified by microarrays was further verified using qPCR gene expression analysis **(** **Fig. 3C** **, Fig. S7)**. In those analyses, mannitol was used as a control to assess possible effects of Xyl on gene expression. Generally, Xyl had only small effect on gene expression, with the exception of *ERF104* and *TPS9*; however, the direction of this effect was the same as for other sugars tested, suggesting that carbon from Xyl can enter a common metabolic pathway for other sugars [19]. For effects of Glc, Fru and Suc, the qPCR analyses confirmed that the expression of the genes was sensitive to either one, two or three sugars, indicating that the microarray data were reliable as to the identification of sugar-responsive genes. However, in several cases, especially for genes responding to a single sugar on microarrays, the qPCR analysis revealed that the genes were also sensitive to one or two other sugars. This was most likely due to higher sensitivity of qPCR when compared to microarray analyses. Indeed, the qPCR-quantified expression of 14 genes correlated linearly to the microarray-determined expression with an average R^2^ of 0.82±0.20 and an overall gene expression ratio ranging from 0.81 to 1.34 (for microarrays) and 0.13 to 4.12 (for qPCR). Thus, the identification of sugar-responsive genes using stringent selection criteria for microarrays was fully confirmed using the more sensitive method. Overall, the qPCR analysis allowed for the identification of genes responding to either all applied sugars, or hexoses only, or Suc and Fru, or Fru only **(** **Fig. 3C** **, Fig. S7)**.

Genes responsive to all three sugars included *bZIP63* (*At5g28770*), *TOR* (*At1g50030*), *At5g22920* (encoding putative RING-type Zinc finger protein), *TPS9* (*At1g23870,* class-II trehalose-P synthase), *XTH30* (*At1g32170,* xyloglucan endotransglucosylase-related protein), *MGD2* (*At5g20410*, type-B monogalactosyl-diacyl-glycerol synthase*)*, and *ERF104* (*At5g61600*, ethylene responsive factor), the latter also Xyl-responsive **(** **Fig. 3C** **, Fig. S7)**. Some of those genes were earlier reported as sugar responsive in studies on whole plants, e.g. expression of *At5g22920* responded to Suc application in *Arabidopsis* seedlings [7] and, along with *TPS9*, to sugar availability during diurnal cycle in intact plants [4], [29]. The qPCR-identified genes responding only to Glc and Fru, but not Suc, included *At2g22080* (encoding unknown protein), *URH1* (*At2g36310*, purine hydrolyzing protein) and *PRKR* (*At3g49160*, pyruvate kinase related) **(** **Fig. 3C** **, Fig. S7)**. Both *URH1* and *At2g22080* have not been previously identified as sugar-responsive, whereas *PRKR* was shown to respond to diurnal changes in *A. thaliana* wt and *pgm1* mutant, and displaying lowest expression during high C-availablity [29]. Finally, based on qPCR analyses, one gene (*At3g25400,* encoding unknown protein) was responsive to Fru and Suc, but not Glc **(Fig. S7)**, and one gene (*At3g57540,* remorin-like) responded to Fru only **(** **Fig. 3C** **)**.

The qPCR approach allowed to verify the effects of specific sugars on expression of selected candidate genes, when compared to the microarrays. For all genes regulated by more than one sugar, as found both by qPCR and microarrays, the effects of various sugars were always in the same direction of expression (up- or down-regulation), although the magnitude of response could differ **(** **Fig. 3C** **, Fig. S7)**. This suggested that different distinct sugar signals for those genes may converge at the transcriptional level. Alternatively, the results may also imply rapid sugar inter-conversions/metabolism, resulting in indirect effects. In addition, the results could not differentiate between possible spatial sites of sugar perception for each gene, i.e. whether outside or inside the cell, nor provide a clue about any signaling pathway transducing the sugar signal for a given gene.

### Dissecting sugar signals and perception sites with sugar analogues

Functional analysis of the signalling by distinct sugars was performed to verify whether gene expression occurred independently from the impact of the sugar molecule as an energy source, and/or resulted from its rapid inter-conversion into another sugar. Non-metabolizable sugar analogues for Glc and Suc were supplied at 1 mM for 1 h to Xyl-adapted cell suspensions. The Glc analogues included L-Glc, which cannot be efficiently transported into the cell, thus allowing for the identification of Glc signaling perceived outside of the cell [5]; and 2-deoxyglucose (2Dog) which is phosphorylated to a dead-end product by hexokinase (HXK), thus allowing for identification of genes which are regulated via a HXK-dependent pathway [1], [5]. The Suc analogues included palatinose (Pal) and turanose (Tur), with only Tur able to enter the plant cells [30], [31]. These compounds were therefore used to dissect gene responsiveness to a specific sugar and in relation to its site of perception (outside and/or inside the cells). As no effective Fru analogues have been reported, an absence of gene response to all the above-mentioned analogues could suggest a Fru-dependent gene expression. In studies with sugar analogues, mannitol was used as a control since, similarly to the analogues, it is non-metabolizable.

**Figure 4:**
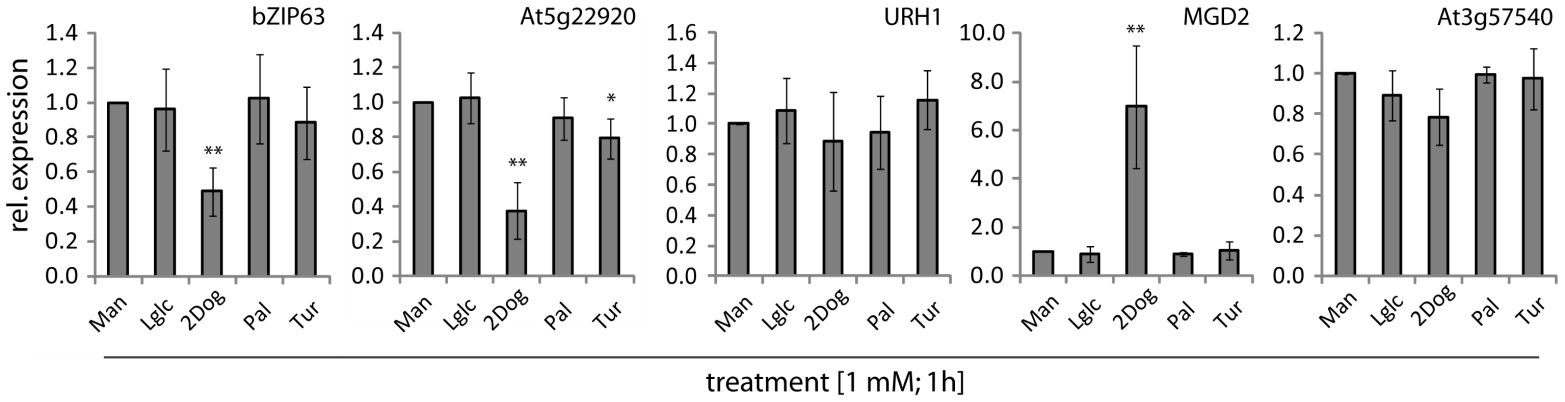
Effects of sugar analogues on expression of selected sugar-responsive genes in 7-d-old Xyl-grown *A.thaliana* cell culture. The analogues were added at 1(mannitol), L-glc (L-glucose), 2Dog (2-deoxyglucose), Pal (palatinose), Tur (turanose). Significance: *t*-test; *α = 0.05, **α = 0.01, n = 5. See also Fig. S8A for examples of other genes.

Among the candidate genes demonstrated by qPCR to respond to all 3 sugars, *bZIP63* and *MGD2* were responsive only to 2Dog; *XTH30* - to L-Glc; *TPS9* and *TOR* - to Tur or Pal, respectively; *At5g22920* - to 2Dog and Tur; *BT2* – to L-Glc and Tur; and *ERF104* - to none of the analogues tested **(** **Fig. 4** **, Fig. S8A)**. The results imply that expression of *bZIP63*, *MGD2* and *At5g22920* involves the action of HXK, while expression of *XTH30* and *BT2* requires an external Glc (both genes) and external Suc (*BT2* only) signal. Expression of *At5g22920* was additionally regulated through a Suc-specific pathway, which requires the uptake of Suc, as shown by the Tur effect **(** **Fig. 4** **)**. A similar Suc-pathway for regulation of gene expression can be proposed for *TPS9*, but not *TOR* expression, as the latter was affected by extracellular Suc (based on Pal effects) **(Fig. S8A)**. The fact that the analogues had no effect on expression of *ERF104* **(Fig. S8A)** suggests a putative signaling by Fru or that the gene is responsive to a downstream metabolite arisen from sugar metabolism.

For genes found by qPCR to respond only to Glc and Fru, but not Suc, sugar analogues had no effect on expression of *URH1* **(** **Fig. 4** **)**, suggesting that the actual signaling molecule is Fru or another signal, which could not be identified with our experimental setup. A change in expression of *At2g22080* and *PRKR* was induced by 2Dog **(Fig. S8A)**, consistent with regulation through a HXK-dependent process. Unexpectedly, Pal affected *PRKR* expression **(Fig. S8A)**, in contrast to the lack of Suc effect on this gene **(Fig. S7)**. This may represent an example of a distinctive signal induction derived from the non-metabolizable sugar analogue (Pal), as reported earlier [32]. Expression of *At3g25400*, which was responsive to Fru and Suc, but not Glc **(Fig. S7)**, was affected by L-Glc and 2Dog **(Fig. S8A)**, suggesting that the Suc effect was indirect, resulting from its hydrolysis to Glc and Fru, but the true nature of the signal is unclear. Finally, *At3g57540* expression, which responded to Fru only **(** **Fig. 3C** **)**, was not affected by the analogues applied **(** **Fig. 4** **)**, suggesting a Fru-dependent pathway, similarly to *URH1*. For all genes where Fru, Glc and 2Dog affected the expression, Fru effects were likely signaled via a Fru-specific pathway, which is distinct from HXK-mediated mechanism, but feeds into the same down-stream regulation pathway [8], [33].

In addition to studies with the above-mentioned sugar analogues, we have also tested effects of 3-*O*-methylglucose (3OMG), a D-Glc analogue, which is transported into plant cells but is metabolized very slowly [34]. This compound, although at first frequently used to identify genes responding to Glc via a HXK-independent pathway (e.g. in [5]), was later proposed to be not perceived as a sugar signal by plants [34] and was ineffective in modulating the expression of over 200 Glc-responsive genes in *Arabidopsis* [35]. In our experimental setup, 3OMG at 1 mM had significant effects on expression of *MGD2*, *TOR* and *PRKR* **(Fig. S8B)**; however, the direction of expression induced by 3OMG was in all three cases opposite to the direction of sugar-induced expression for a given gene. Thus, strikingly, 3OMG behaved as a classical antagonist of Glc effect, suggesting that it interferes with a Glc perception/transduction pathway. It is unclear, however, whether those results are relevant to sugar signaling, given the controversy surrounding the effectiveness of 3OMG in truly reflecting a sugar signal in plants.

Studies with sugar analogues have demonstrated that the cells had the ability to effectively inter-convert exogenously added sugar molecules, and this concerned not only the inter-conversion of Suc to/from Glc and Fru, but also between the two hexoses. The inter-conversions occurred even though the sugars were applied at low concentration (1 mM) and for short time (1 h), i.e. conditions which apparently had little or no significant effect on internal sugar pools **(Fig. S2)**. This suggested that the active quantity of the exogenously applied sugar required to induce a change in gene expression was lower than 1 mM. Nevertheless, using a combination of treatments with a given sugar and a given sugar analogue, we were able to unequivocally pinpoint the true nature of the signalling sugar for a given gene tested. The genes responded rapidly to low concentrations of a given sugar and can, thus, be considered as part of an early response mechanism to the sugar availability for cells otherwise adapted to grow under long-term low carbon conditions.

Altogether, the functional dissection with sugar analogues allowed to identify genes that are specifically regulated not only by one sugar species (e.g. Fru, *At3g57540*), but also by two (Glc and Suc for *At5g22920*) or, for some genes, perhaps even by each of the three sugars, given that we could not unequivocally prove Fru-specific effects. The mechanisms of a given sugar signaling may also differ, with Suc or Glc sensed outside or inside of the cell, with some of the Glc effects signaled via the HXK-dependent or HXK-independent pathways. In **Table S2**, we have summarized the results of sugar- and sugar analogue-dependent expression of selected genes and have listed the identified sugar-specific gene responses and sites of sugar sensing for each gene.

### Expression of sugar-responsive genes *in planta*

**Figure 5:**
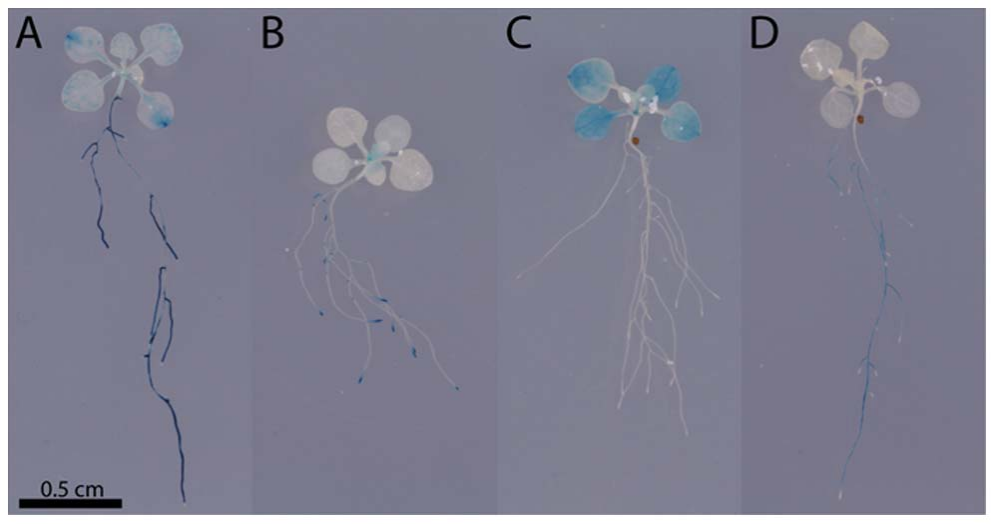
The expression of selected sugar-responsive genes *in planta* is not restricted to a specific tissue or cell type, as found by GUS expression analyses of four promoter::reporter lines for *bZIP63* (A), *TOR* (B), *MRS2.11* (C) and *URH1* (D) in 13-d-old *Arabidopsis* seedlings.

To assess the biological relevancy of the use of habituated cell culture in identifying sugar-responsive genes, expression of selected genes was studied first in transgenic plants transformed with promoter-GUS reporter. GUS-expression analyses for *bZIP63*, *MRS2.11*, *URH1* and *TOR* revealed the cell/tissue/organ-specific expression patterns of each gene: *bZIP63* - in roots, cotyledon meristems and leaf vasculature, *TOR* – in meristems of both apical shoot and side roots, *MRS2.11* - mainly in leaf mesophyll cells, and *URH1* - in roots only **(** **Fig. 5** **)**. A similar distribution of GUS-expression for those genes was reported earlier [36]–[39]. The expression of the genes in whole seedlings was not restricted to one specific tissue- or cell-type, but represented a variety of tissues/cells, both autotrophic and heterotrophic. This was supported by a more detailed Genevestigator-assisted analysis of tissue-dependent expression which included - besides *bZIP63*, *MRS2.11*, *URH1* and *TOR* – also other genes that were selected for qPCR studies **(Fig. S5B)**. Thus, the identification of sugar-responsive genes in cell culture was not biased toward genes preferentially expressed in any specific type of cells/tissues.

**Figure 6:**
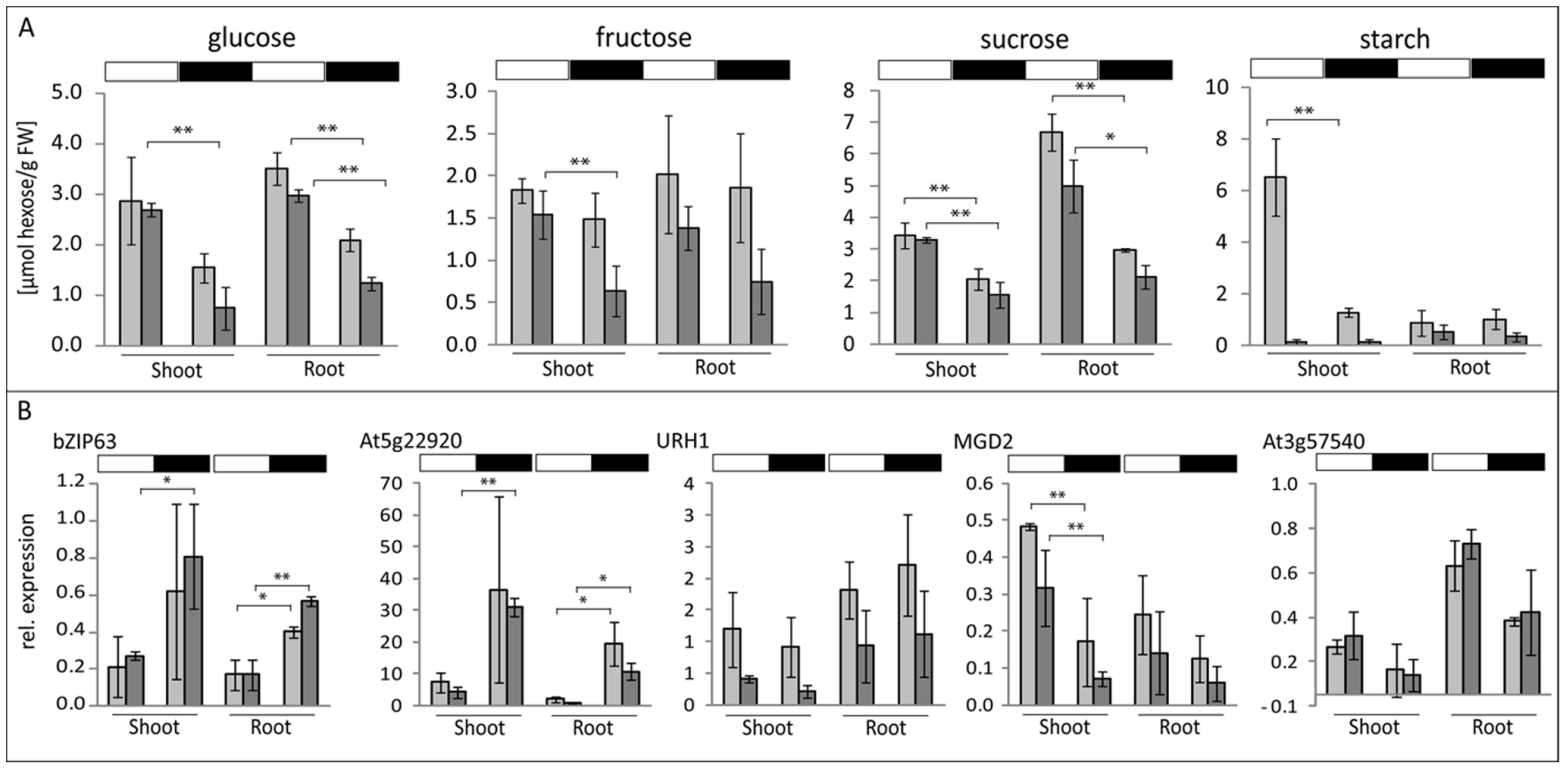
Endogenous sugar contents (A) and the expression of cell-culture-selected sugar-responsive genes (B) respond to light/dark conditions in intact plants. Shoots and roots from 13-d-old *A. thaliana* seedlings of wt (light bar) and *pgm1* (dark bar), grown at 16/8 h photoperiod, were harvested at the stages of high (end of 16 h day, white box) and low (end of 6 h extended night, black box) carbon availability. Significance: *t*-test; *α = 0.05, **α = 0.01, n = 3. See also Fig. S9 for examples of other genes.

In subsequent studies, using qPCR, we assessed whether the genes identified with the cell culture system were also sugar-responsive in intact *Arabidopsis* plants which experienced changes in internal sugar concentrations. When wt plants and the starch-deficient *pgm1* mutant, both grown in the 16 h light/8 h darkness photoperiod, were exposed to a 6 h extended night conditions, the endogenous soluble sugar contents decreased in comparison to the end of light conditions **(** **Fig. 6A** **)**. Changes in sugar contents were accompanied by changes in expression of sugar-responsive genes; this was observed in both shoots and roots for both wt and *pgm1* seedlings **(** **Fig. 6B** **, Fig. S9)**. Since the *pgm1* has little or no starch **(** **Fig. 6A** **)**, the similar expression patterns of sugar-responsive genes in wt and *pgm1* plants suggested that their expression was independent of the formation of starch or the availability of starch as backup energy source. Generally, we observed little or no difference in the contents of soluble sugars between the *pgm1* mutant and wt plants **(** **Fig. 6A**). Even though these results are contrary to observations published earlier, it has been highlighted that the ratio of soluble sugar content in the *pgm1* versus wt plants is dependent on growth conditions, especially the length of the photoperiod (reviewed in [40]). In contrast to other studies, the seedlings were germinated and grown *in vitro* under long-day conditions (16 h light/8 h dark regime) which resulted in a comparable phenotype of *pgm1* and wt. In addition, the samples were harvested at an early developmental stage (after 13 days), a time point where the difference in the soluble sugar contents between *pgm1* and wt is less established.

For a given gene, the direction of the change of expression (increase or decrease) was as observed in the sugar-treated cell culture. Thus, *BT2, bZIP63, At5g22920*, and *TPS9* were significantly up-regulated by dark conditions (low sugar content), whereas *MGD2* and *MRS2.11* were up-regulated in the light (high sugar content) **(** **Fig. 6B** **, Fig. S9)**. Other tested genes, with the possible exception of *URH1*, followed the same trend, even though the results were not always statistically significant. Similar to the GUS-expression patterns observed in transgenic plants **(** **Fig. 5** **)**, the highest *MRS1* expression was in shoots, while *URH1* was mainly expressed in roots. Both *bZIP63* and *TOR* were expressed about equally in shoots and roots **(** **Fig. 6B** **, Fig. S9)**.

Overall, the results strongly suggest that the photoperiod-mediated changes in endogenous sugar contents *in planta* play a significant, if not decisive, role in regulating expression of the selected genes. The identities of effective sugars could not be inferred from those data, as contents of all sugars changed in a similar way during the light/dark conditions **(** **Fig. 6A** **)**, and we could not control sugar contents within each specific cell-type within the organ studied (shoot vs. root). However, for the first time, we can rationally infer the true identities of the effective sugars and specific sugar-signaling, based on the data obtained from the habituated cell cultures.

### Habituated cells as a model system to study sugar signalling

In plants, each cell type either produces its own sugars and/or imports them from other cells. As sugars are the main energy source and provide the backbone for all carbon-containing compounds, the proper functioning of the whole plant depends on a highly coordinated network of sugar molecules being produced and metabolized, exported and imported, both between adjacent cells and via long-distance transport through the phloem. This adds to the complexity of studies on sugar-signaling in whole plants, where each cell type or tissue may face different sugar concentrations and may have distinct cell/tissue-specific mechanisms modulating expression of sugar-responsive genes. Another difficulty concerns deciphering between the direct and indirect effects of sugars, as an increase in sugar content stimulates both auxin and ABA synthesis [28], [41], whereas sugar starvation inhibits gibberellin production [42]. In intact plants, studies on the impact of sugars and hormones in modulating gene expression have to take into account the respective weight/impact of their cross-talk in each cell/tissue-type within the organism. These limitations do not apply when using the habituated cell culture system, which is moreover highly versatile with respect to requirements for carbon source **(Fig. S1)**.

Sugar-responsiveness of a given gene depends on availability of specific sugar signal(s), but it may also depend on developmental status of different cells/tissues. In the present study, sugar-responsiveness of the identified genes, although determined within the context of a heterotrophic cell culture, was not restricted to a distinct cell, tissue or organ in whole plants **(** **Fig. 5** **, Fig. S5)**. Moreover, expression of those genes was correlated with internal changes in sugar concentration in whole plants during diurnal cycle, but the magnitude of expression was dependent on a given organ **(** **Fig. 6** **)**. This strongly suggests that the results obtained with the habituated cell culture are of high relevance to studies on sugar signaling in whole plants. Aside from other advantages, the culture represents an ideal system in which exogenous supply of specific sugar(s) and/or morphogen(s) and/or cell differentiation status can be controlled by the experimenter. The system can also be used to study effects and mechanisms of action of any other signaling compounds (e.g. a plant hormone), with or without the sugar cross-talk.

## Supporting Information

Figure S1 **Effects of various C-sources on growth of** ***A. thaliana*** **cell culture (A) and specific effects of Xyl (B) and (C) on cell length and general phenotype of the cultured cells.**
(DOCX)

Figure S2 **Effects of Suc concentration and induction time on the intracellular contents of soluble sugars in** ***A. thaliana*** **cell culture.**
(DOCX)

Figure S3 **The global gene expression response to exogenously applied sugars overlapp between** ***A.thaliana*** **cell culture and seedlings.**
(DOCX)

Figure S4 **The effect of the treatment of plant material with varying Suc and Glc concentrations on the expression of 290 identified genes (A) and of the 14 selected genes (B).**
(DOCX)

Figure S5 **Distribution of the expression of 290 identified genes (A) and of the 14 selected genes (B) in various tissues/organs.**
(DOCX)

Figure S6 **Functional classification of 290 identified genes.**
(DOCX)

Figure S7 **Defined sets of sugars trigger significant changes in gene expression of selected sugar-responsive genes in 7-d-old** ***A.thaliana*** **cell culture.**
(DOCX)

Figure S8 **Effects of sugar analogues on expression of selected sugar-responsive genes in 7-d-old Xyl-grown** ***A.thaliana*** **cell culture.**
(DOCX)

Figure S9 **The expression of cell-culture-selected sugar-responsive genes responds to light/dark conditions in intact plants.**
(DOCX)

Table S1 **Information on primer sequences used for qPCR analyses conducted within the study.**
(DOCX)

Table S2 **Summary of sugar regulation of selected genes in** ***A.thaliana*** **cell culture.**
(DOCX)

Dataset S1 **Comparison of a set of sugar responsive genes identified in the present study (habituated cell culture) with sets of genes identified for whole plants in studies by Usadel** ***et al.*** **** [4] **, Bläsing** ***et al*** **.** [25] **and Osuna** ***et al.*** **** [7] **.**
(XLSX)

## References

[1] Koch KE (1996) Carbohydrate-modulated gene expression in plants. Annu Rev Plant Physiol Plant Mol Biol 47: 509–540. 15012299 10.1146/annurev.arplant.47.1.509

[2] Rolland F, Baena-Gonzalez E, Sheen J (2006) Sugar sensing and signaling in plants: conserved and novel mechanisms. Annu Rev Plant Biol 57: 675–709. 16669778 10.1146/annurev.arplant.57.032905.105441

[3] Smith AM, Stitt M (2007) Coordination of carbon supply and plant growth. Plant Cell Environ 30: 1126–1149. 17661751 10.1111/j.1365-3040.2007.01708.x

[4] Usadel B, Bläsing OE, Gibon Y, Retzlaff K, Höhne M, et al (2008) Global transcript levels respond to small changes of the carbon status during progressive exhaustion of carbohydrates in *Arabidopsis* rosettes. Plant Physiol 146: 1834–1861. 18305208 10.1104/pp.107.115592 PMC2287354

[5] Jang JC, Sheen J (1994) Sugar sensing in higher plants. Plant Cell 6: 1665–1679. 7827498 10.1105/tpc.6.11.1665 PMC160552

[6] Contento AL, Kim SJ, Bassham DC (2004) Transcriptome profiling of the response of *Arabidopsis* suspension culture cells to sucrose starvation. Plant Physiol 135: 2330–2347. 15310832 10.1104/pp.104.044362 PMC520801

[7] Osuna D, Usadel B, Morcuende R, Gibon Y, Bläsing OE, et al (2007) Temporal responses of transcripts, enzyme activities and metabolites after adding sucrose to carbon-deprived *Arabidopsis* seedlings. Plant J 49: 463–491. 17217462 10.1111/j.1365-313X.2006.02979.x

[8] Li P, Wind JJ, Shi X, Zhang H, Hanson J, et al (2011) Fructose sensitivity is suppressed in *Arabidopsis* by the transcription factor ANAC089 lacking the membrane-bound domain. Proc Natl Acad Sci USA 108: 3436–3441. 21300879 10.1073/pnas.1018665108 PMC3044370

[9] Ciereszko I, Johansson H, Kleczkowski LA (2005) Interactive effects of phosphate deficiency, sucrose and light/dark conditions on gene expressionof UDP-glucose pyrophosphorylase in *Arabidopsis.* . J Plant Physiol 162: 343–353. 15832687 10.1016/j.jplph.2004.08.003

[10] Vaughn MW, Harrington GN, Bush DR (2002) Sucrose-mediated transcriptional regulation of sucrose symporter activity in the phloem. Proc Natl Acad Sci U*SA* 99: 10876–10880. 12149483 10.1073/pnas.172198599 PMC125066

[11] Lloyd JC, Zakhleniuk OV (2004) Responses of primary and secondary metabolism to sugar accumulation revealed by microarray expression analysis of the *Arabidopsis* mutant, *pho3* . J Exp Bot 55: 1221–1230. 15133053 10.1093/jxb/erh143

[12] Chaudhuri B, Hörmann F, Lalonde S, Brady SM, Orlando DA, et al (2008) Protonophore- and pH-insensitive glucose and sucrose accumulation detected by FRET nanosensors in *Arabidopsis* root tips. Plant J 56: 948–962. 18702670 10.1111/j.1365-313X.2008.03652.x PMC2752219

[13] Kawasaki H, Mizuseki K, Nishikawa S, Kaneko S, Kuwana Y, et al (2000) Induction of midbrain dopaminergic neurons from ES cells by stromal cell-derived inducing activity. Neuron 28: 31–40. 11086981 10.1016/s0896-6273(00)00083-0

[14] Yamashita J, Itoh H, Hirashima M, Ogawa M, Nishikawa S, et al (2000) Flk-1-positive cells derived from embryonic stem cells serve as vascular progenitor. Nature 408: 92–96. 11081514 10.1038/35040568

[15] Ito Y, Nakanomyo I, Motose H, Iwamoto K, Sawa S, et al (2006) Dodeca-CLE peptides as suppressors of plant stem cell. Science 313: 842–845. 16902140 10.1126/science.1128436

[16] Sugimoto K, Gordon SP, Meyerowitz EM (2011) Regeneration in plants and animals: dedifferentiation, transdifferentiation, or just differentiation? Trends Cell Biol 21: 212–218. 21236679 10.1016/j.tcb.2010.12.004

[17] Pesquet E, Korolev AV, Calder G, Lloyd CW (2010) The Microtubule-Associated Protein AtMAP70-5 regulates secondary wall patterning in *Arabidopsis* wood cells. Curr Biol 20: 744–749. 20399097 10.1016/j.cub.2010.02.057

[18] Hruz T, Laule O, Szabo G, Wessendorp F, Bleuler S, et al (2008) Genevestigator V3: a reference expression database for the meta-analysis of transcriptomes. Adv Bioinf 2008: 420747. 10.1155/2008/420747 PMC2777001 19956698

[19] Carpita NC, Brown RA, Weller KM (1982) Uptake and metabolic fate of glucose, arabinose, and xylose by *Zea mays* coleoptiles in relation to cell wall synthesis. Plant Physiol 69: 1173–1180. 16662366 10.1104/pp.69.5.1173 PMC426380

[20] Kato A, Tohoyama H, Joho M, Inouhe M (2007) Different effects of galactose and mannose on cell proliferation and intracellular soluble sugar levels in *Vigna angularis* suspension cultures. J Plant Res 120: 713–9. 17917698 10.1007/s10265-007-0117-9

[21] Stevenson CC, Harrington GN (2009) The impact of supplemental carbon sources on *Arabidopsis thaliana* growth, chlorophyll content and anthocyanin accumulation. Plant Growth Regul 59: 255–271.

[22] Stitt M, Gibon Y, Lunn JE, Piques M (2007) Multilevel genomics analysis of carbon signalling during low carbon availability: coordinating the supply and utilisation of carbon in a fluctuating environment. Funct Plant Biol 34: 526–549. 10.1071/FP06249 32689382

[23] Mandadi KK, Misra A, Ren S, McKnight TD (2009) BT2, a BTB protein, mediates multiple responses to nutrients, stresses, and hormones in *Arabidopsis.* . Plant Physiol 150: 1930–1939. 19525324 10.1104/pp.109.139220 PMC2719139

[24] Hannah MA, Heyer AG, Hincha DK (2005) A global survey of gene regulation during cold acclimation in *Arabidopsis thaliana* . PLoS Genetics 1(2): e26 doi:10.1371/journal.pgen.0010026 16121258 10.1371/journal.pgen.0010026 PMC1189076

[25] Bläsing OE, Gibon Y, Günther M, Höhne M, Morcuende R, et al (2005) Sugars and circadian regulation make major contributions to the global regulation of diurnal gene expression in *Arabidopsis* . Plant Cell 17: 3257–3281. 16299223 10.1105/tpc.105.035261 PMC1315368

[26] Robaglia C, Thomas M, Meyer C (2012) Sensing nutrient and energy status by SnRK1 and TOR kinases. Curr. Opin. Plant Biol 15: 301–307. 22305521 10.1016/j.pbi.2012.01.012

[27] Xiong Y, McCormack M, Li L, Hall Q, Xiang C, et al (2013) Glucose-TOR signalling reprograms the transcriptome and activates meristems. Nature 496: 181–186. 23542588 10.1038/nature12030 PMC4140196

[28] Matiolli CC, Pires Tomaz J, Turqueto Duarte G, Prado FM, Del Bem LE, et al (2011) The *Arabidopsis* bZIP gene *AtbZIP63* is a sensitive integrator of transient abscisic acid and glucose signals. Plant Physiol 157: 692–705. 21844310 10.1104/pp.111.181743 PMC3192551

[29] Gibon Y, Usadel B, Blaesing OE, Kamlage B, Hoehne M, et al (2006) Integration of metabolite with transcript and enzyme activity profiling during diurnal cycles in *Arabidopsis* rosettes. BMC Genome Biol 7(8): R76. 10.1186/gb-2006-7-8-r76 PMC1779593 16916443

[30] Chandran D, Reinders A, Ward JM (2003) Substrate specificity of the *Arabidopsis thaliana* sucrose transporter *AtSUC2* . J Biol Chem 278: 44320–44325. 12954621 10.1074/jbc.M308490200

[31] Fernie AR, Roessner U, Geigenberger P (2001) The sucrose analogue palatinose leads to a stimulation of sucrose degradation and starch synthesis when supplied to discs of growing potato tubers. Plant Physiol 125: 1967–1977. 11299376 10.1104/pp.125.4.1967 PMC88852

[32] Sinha AK, Hofmann MG, Römer U, Köckenberger W, Elling L, et al (2002) Metabolizable and non-metabolizable sugars activate different signal transduction pathways in tomato. Plant Physiol 128: 1480–1489. 11950996 10.1104/pp.010771 PMC154275

[33] Cho YH, Yoo SD (2011) Signaling role of fructose mediated by FINS1/FBP in *Arabidopsis thaliana* . PLoS Genetics 7: e1001263. 21253566 10.1371/journal.pgen.1001263 PMC3017112

[34] Cortès S, Gromova M, Evrard A, Roby C, Heyraud A, et al (2003) In plants, 3-*O*-methylglucose is phosphorylated by hexokinase but not perceived as a sugar. Plant Physiol 131: 824–837. 12586906 10.1104/pp.010538 PMC166858

[35] Villadsen D, Smith SM (2004) Identification of more than 200 glucose-responsive *Arabidopsis* genes none of which responds to 3-*O*-methylglucose or 6-deoxyglucose. Plant Mol Biol 55: 467–477. 15604693 10.1007/s11103-004-1050-0

[36] Menand B, Desnos T, Nussaume L, Berger F, Bouchez D, et al (2002) Expression and disruption of the *Arabidopsis TOR* (target of rapamycin) gene. Proc Natl Acad Sci USA 99: 6422–6427. 11983923 10.1073/pnas.092141899 PMC122964

[37] Gebert M, Meschenmoser K, Svidová S, Weghuber J, Schweyen R, et al (2009) A root-expressed magnesium transporter of the MRS2/MGT gene family in *Arabidopsis thaliana* allows for growth in low-Mg^2+^ environments. Plant Cell 21: 4018–4030. 19966073 10.1105/tpc.109.070557 PMC2814501

[38] Jung B, Flörchinger M, Kunz HH, Traub M, Wartenberg R, et al (2009) Uridine-ribohydrolase is a key regulator in the uridine degradation pathway of *Arabidopsis* . Plant Cell 21: 876–891. 19293370 10.1105/tpc.108.062612 PMC2671717

[39] Weltmeier F, Rahmani F, Ehlert A, Dietrich K, Schutze K, et al (2009) Expression patterns within the *Arabidopsis* C/S1 bZIP transcription factor network: availability of heterodimerization partners controls gene expression during stress response and development. Plant Mol Biol 69: 107–119. 18841482 10.1007/s11103-008-9410-9 PMC2709229

[40] Caspar T (1994) Genetic dissection of the biosynthesis, degradation, and biological functions of starch. In: Meyerowitz EM, Somerville CR (eds.), Arabidopsis. Cold Spring Harbor Laboratory Press. 913–936.

[41] Sairanen I, Novák O, Pěnčík A, Ikeda Y, Jones B, et al (2012) Soluble carbohydrates regulate auxin biosynthesis via PIF proteins in *Arabidopsis* . Plant Cell 24: 4907–4916. 23209113 10.1105/tpc.112.104794 PMC3556965

[42] Paparelli E, Parlanti S, Gonzali S, Novi G, Mariotti L, et al (2013) Nighttime sugar starvation orchestrates gibberellin biosynthesis and plant growth in *Arabidopsis* . Plant Cell 25: 3760–3769. 24096343 10.1105/tpc.113.115519 PMC3877829

