# Supplementary material for: Functional Dissection of Sugar Signals Affecting Gene Expression in Arabidopsis thaliana
Source: PLoS One. 2014 Jun 20;9(6):e100312. doi: 10.1371/journal.pone.0100312 (PMC4065033; doi:10.1371/journal.pone.0100312)
Supplement: Figure S1 — Effects of various C-sources on growth of A. thaliana cell culture (A) and specific effects of Xyl (B) and (C) on cell length and general phenotype of the cultured cells. (DOCX) [file pone.0100312.s001.docx]

**
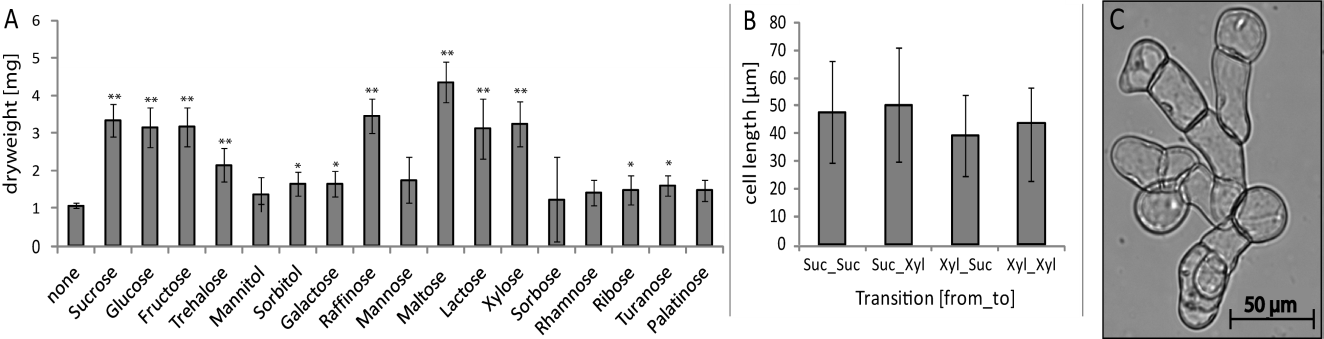
**

**Fig.S1.** Effects of various C-sources on growth of *A. thaliana* cell culture **(A)** and specific effects of Xyl **(B and C)** on cell length and general phenotype of the cultured cells. In **(A)**, the habituated Suc-grown cell culture was transferred into 1x MS media supplemented with 18 different C-sources. The increase in biomass was subsequently analyzed by measurement of the dry weight of cells within 1 ml cell suspension. Sugars which led to a significant increase in biomass were Suc, Glc, Fru, trehalose, raffinose, maltose, lactose and Xyl. In (**B)** and (**C**), cell length and general appearance (shown here for Xyl-grown cells) showed no significant differences between Xyl- and Suc-grown cells. In all cases, error bars represent the standard deviation calculated from 3 biological repeats. Significance: *t*-test; * α=0.05, ** α=0.01.
