# Supplementary material for: Functional Dissection of Sugar Signals Affecting Gene Expression in Arabidopsis thaliana
Source: PLoS One. 2014 Jun 20;9(6):e100312. doi: 10.1371/journal.pone.0100312 (PMC4065033; doi:10.1371/journal.pone.0100312)
Supplement: Figure S2 — Effects of Suc concentration and induction time on the intracellular contents of soluble sugars in A. thaliana cell culture. (DOCX) [file pone.0100312.s002.docx]

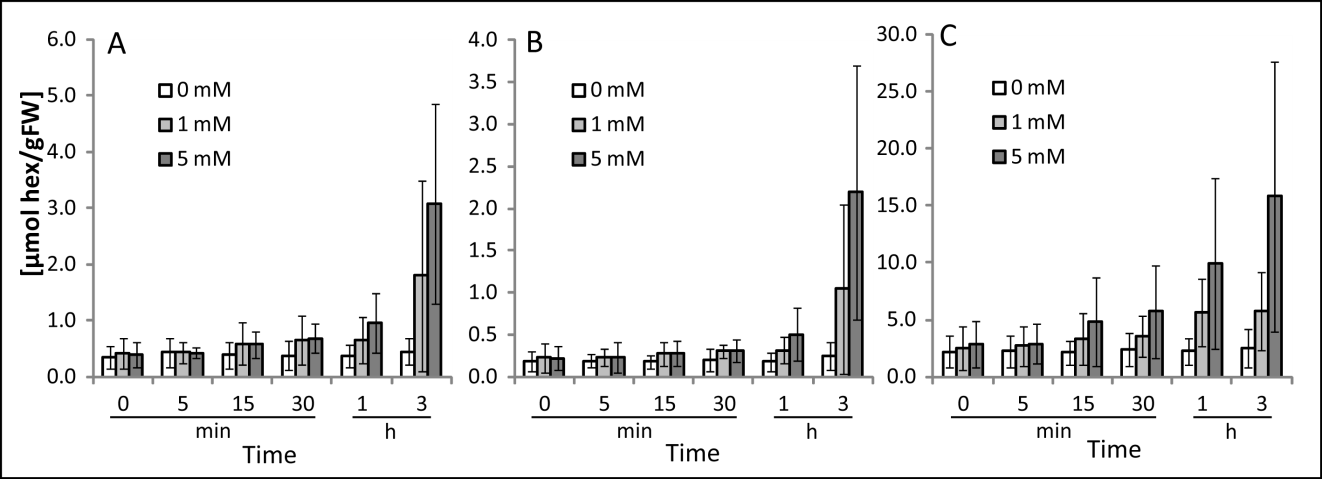


**Fig.S2**. Effects of Suc concentration and induction time on the intracellular contents of soluble sugars in *A.thaliana* cell culture. The induction of 7-d-old Xyl-grown cell culture with 0, 1 and 5 mM Suc for 0, 5, 15, 30 min and 1 and 3 h led to detectable increases of internal concentrations of Glc **(A)**, Fru **(B)** and Suc **(C)** after 1 to 3 h. After 3 h of induction, there were significant differences between 0 and 5 mM Suc treatment. Error bars represent standard deviation of 5 biological repeats.
