# Supplementary material for: Functional Dissection of Sugar Signals Affecting Gene Expression in Arabidopsis thaliana
Source: PLoS One. 2014 Jun 20;9(6):e100312. doi: 10.1371/journal.pone.0100312 (PMC4065033; doi:10.1371/journal.pone.0100312)
Supplement: Figure S3 — The global gene expression response to exogenously applied sugars overlapp between A.thaliana cell culture and seedlings. (DOCX) [file pone.0100312.s003.docx]

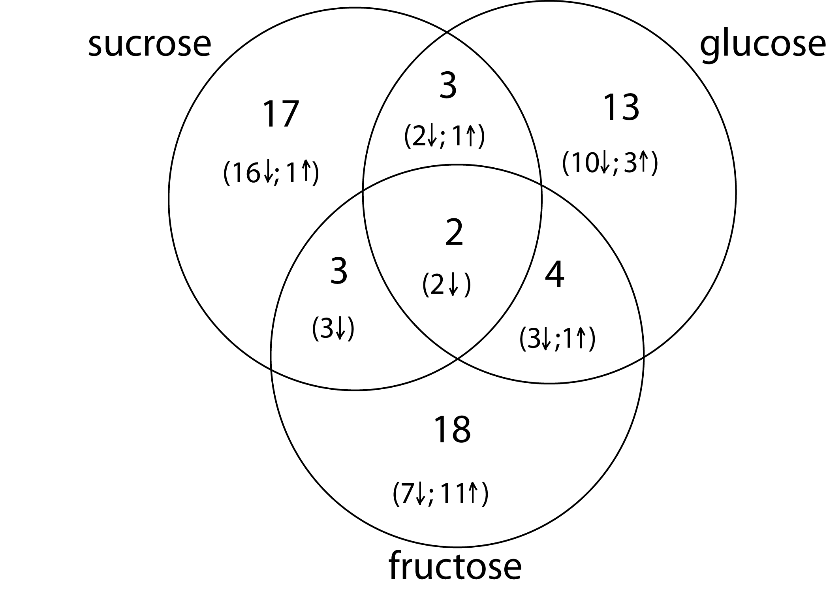


**Fig. S3**. The global gene expression response to exogenously applied sugars overlapp between *A.thaliana* cell culture and seedlings. Comparing the genes identified in this study with the genes identified by Osuna *et al.* (2007) revealed an overlap of 60 genes. Among those were genes responsive to each one of the 7 sub-sets: Suc/Glc/Fru, Suc/Glc, Glc/Fru, Suc/Fru, Glc, Suc, or Fru.
