# Supplementary material for: Functional Dissection of Sugar Signals Affecting Gene Expression in Arabidopsis thaliana
Source: PLoS One. 2014 Jun 20;9(6):e100312. doi: 10.1371/journal.pone.0100312 (PMC4065033; doi:10.1371/journal.pone.0100312)
Supplement: Figure S4 — The effect of the treatment of plant material with varying Suc and Glc concentrations on the expression of 290 identified genes (A) and of the 14 selected genes (B). (DOCX) [file pone.0100312.s004.docx]

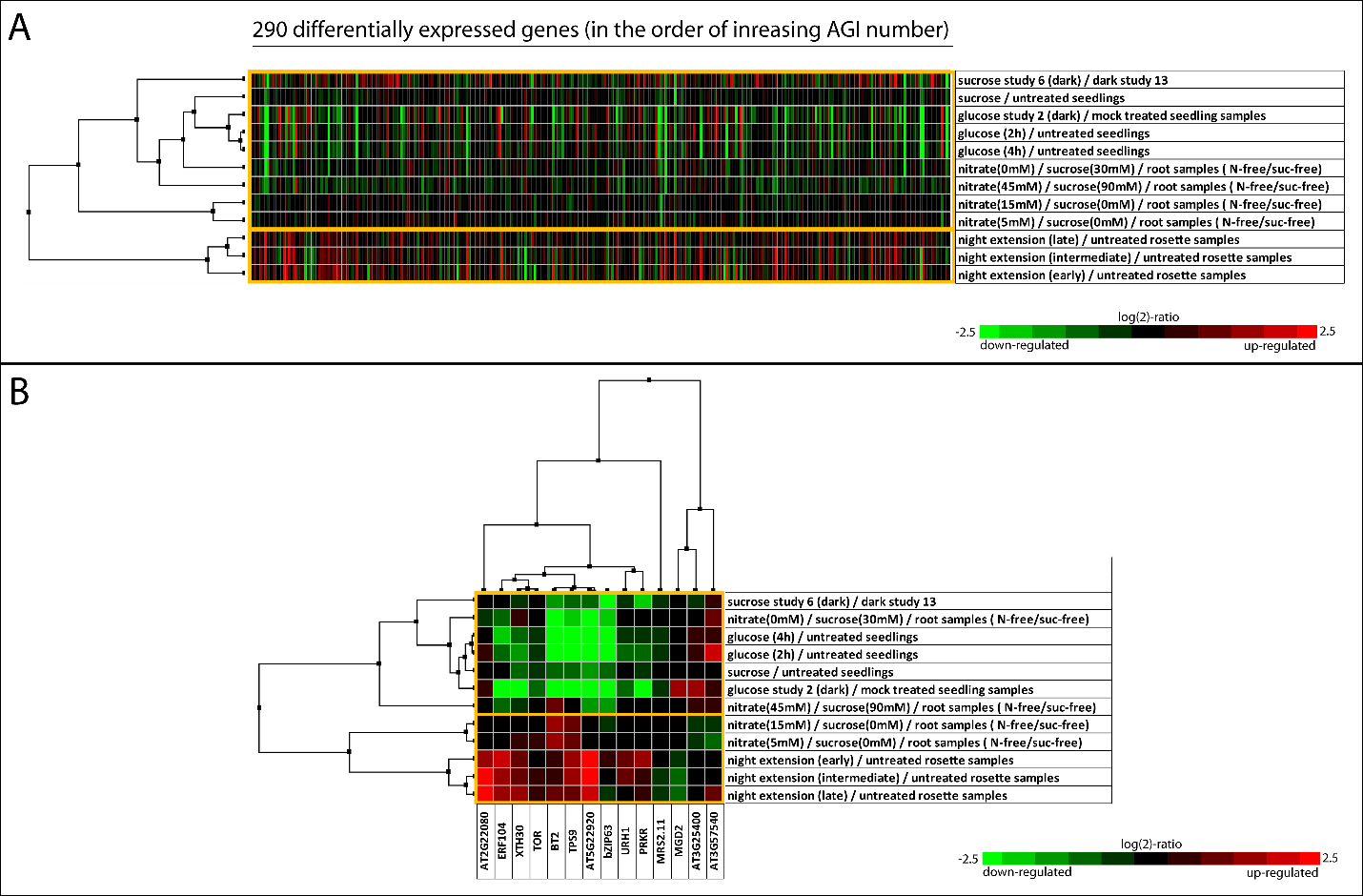


**Fig. S4.** The effect of the treatment of plant material with varying Suc and Glc concentrations on the expression of 290 identified genes **(A)** and of the 14 selected genes **(B)**. The analysis was conducted with Genevestigator using hierarchical clustering based on the Pearson correlation of the expression profiles (represented as log(2)-ratios). The following experiments were selected: AT-0006; 0014; 0015; 0056; 0133; 0199; 0209; 0281; 0639; 0650. Only data from wt samples were used.
