# Supplementary material for: Functional Dissection of Sugar Signals Affecting Gene Expression in Arabidopsis thaliana
Source: PLoS One. 2014 Jun 20;9(6):e100312. doi: 10.1371/journal.pone.0100312 (PMC4065033; doi:10.1371/journal.pone.0100312)
Supplement: Figure S5 — Distribution of the expression of 290 identified genes (A) and of the 14 selected genes (B) in various tissues/organs. (DOCX) [file pone.0100312.s005.docx]

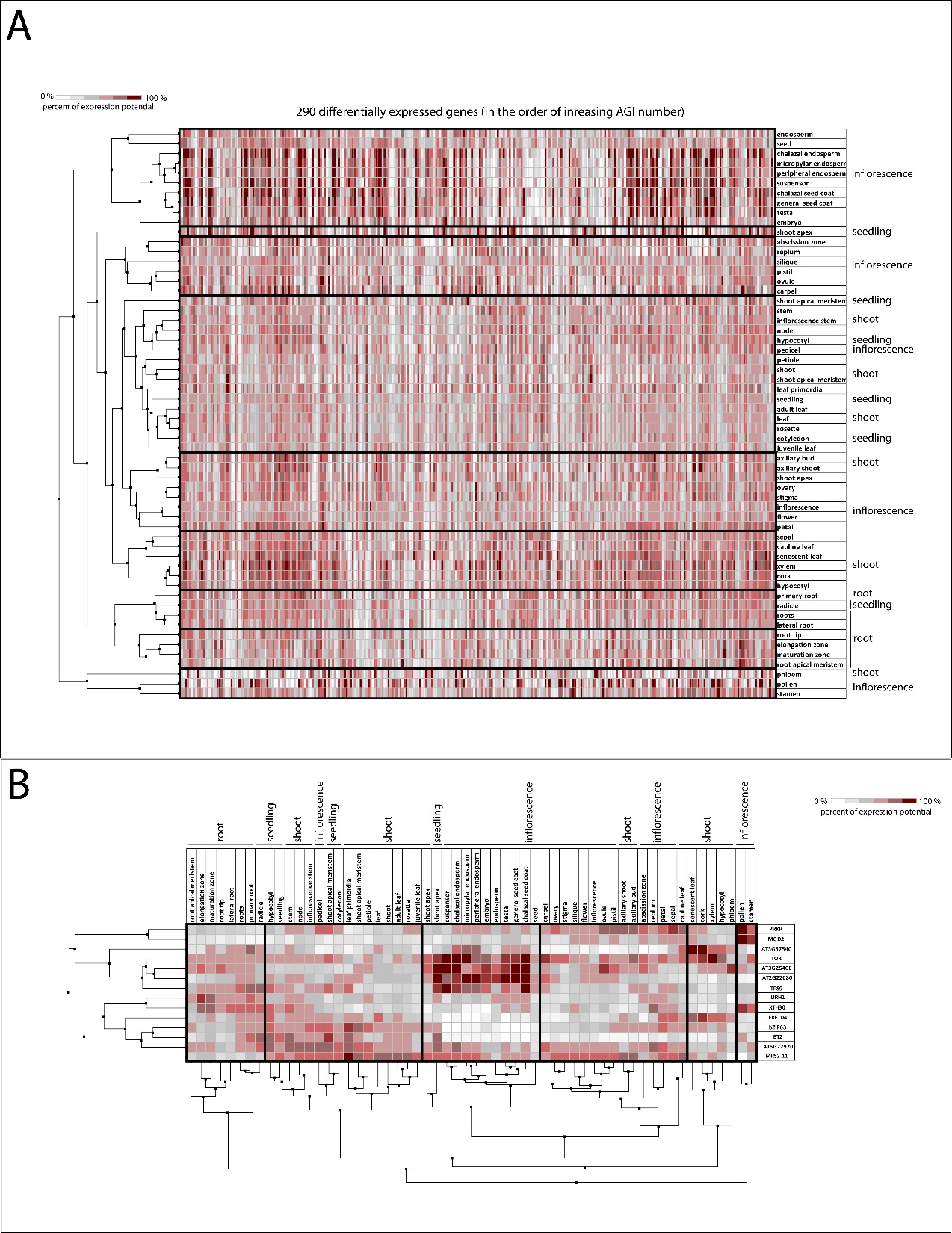


**Fig. S5.** Distribution of the expression of 290 identified genes **(A)** and of the 14 selected genes **(B)** in various tissues/organs. The analysis was conducted with Genevestigator using hierarchical clustering based on the Pearson correlation of the expression profiles.
