# Supplementary material for: Functional Dissection of Sugar Signals Affecting Gene Expression in Arabidopsis thaliana
Source: PLoS One. 2014 Jun 20;9(6):e100312. doi: 10.1371/journal.pone.0100312 (PMC4065033; doi:10.1371/journal.pone.0100312)
Supplement: Figure S6 — Functional classification of 290 identified genes. (DOCX) [file pone.0100312.s006.docx]

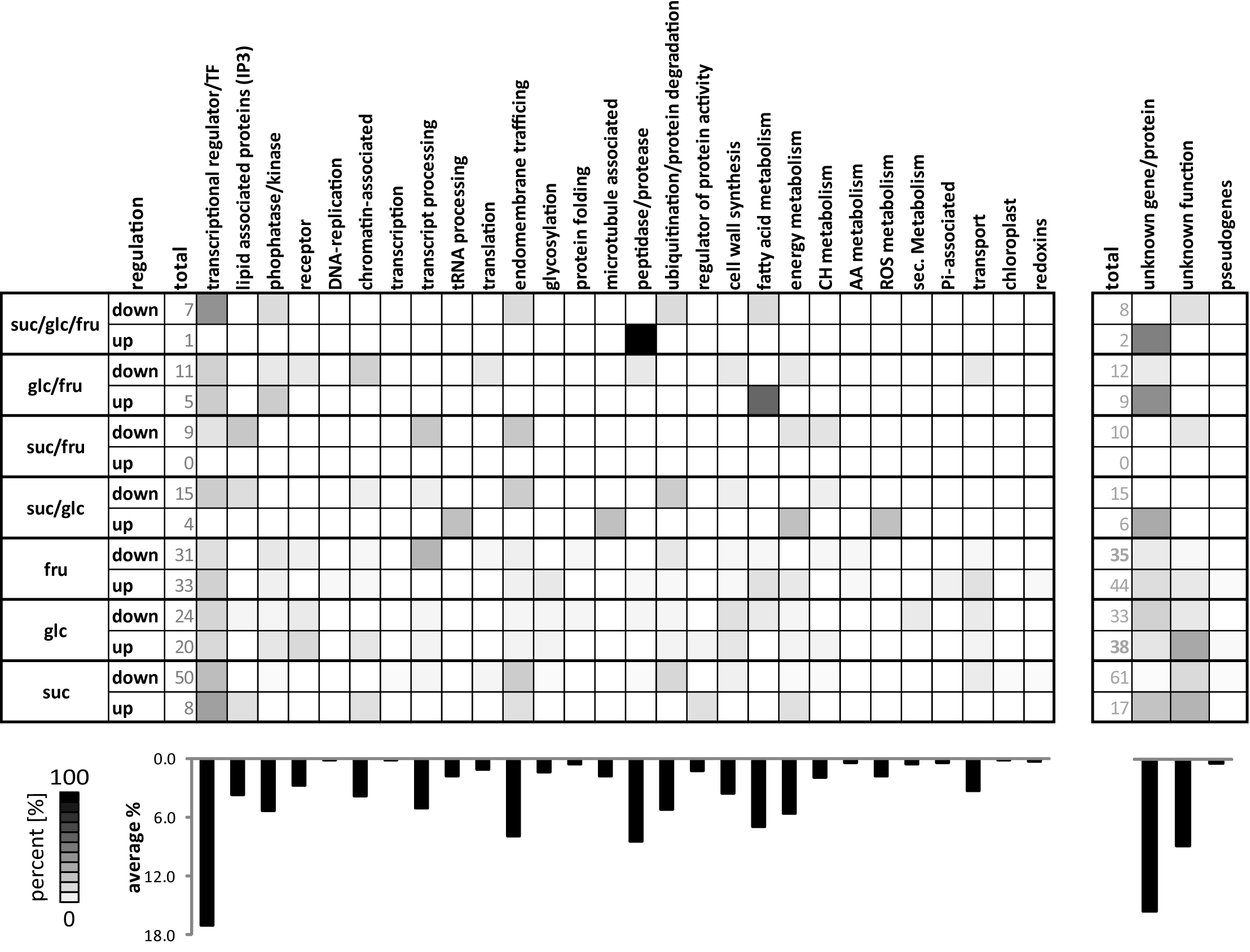


**Fig.S6.** Functional classification of 290 identified genes. Based on GO annotation and literature knowledge, the 290 genes were divided into functional classes. The percentage of appearance of genes in each class was calculated based on the total amount of genes within one sub-group (excluding the unknown and pseudogenes).
