# Supplementary material for: Functional Dissection of Sugar Signals Affecting Gene Expression in Arabidopsis thaliana
Source: PLoS One. 2014 Jun 20;9(6):e100312. doi: 10.1371/journal.pone.0100312 (PMC4065033; doi:10.1371/journal.pone.0100312)
Supplement: Figure S7 — Defined sets of sugars trigger significant changes in gene expression of selected sugar-responsive genes in 7-d-old A.thaliana cell culture. (DOCX) [file pone.0100312.s007.docx]

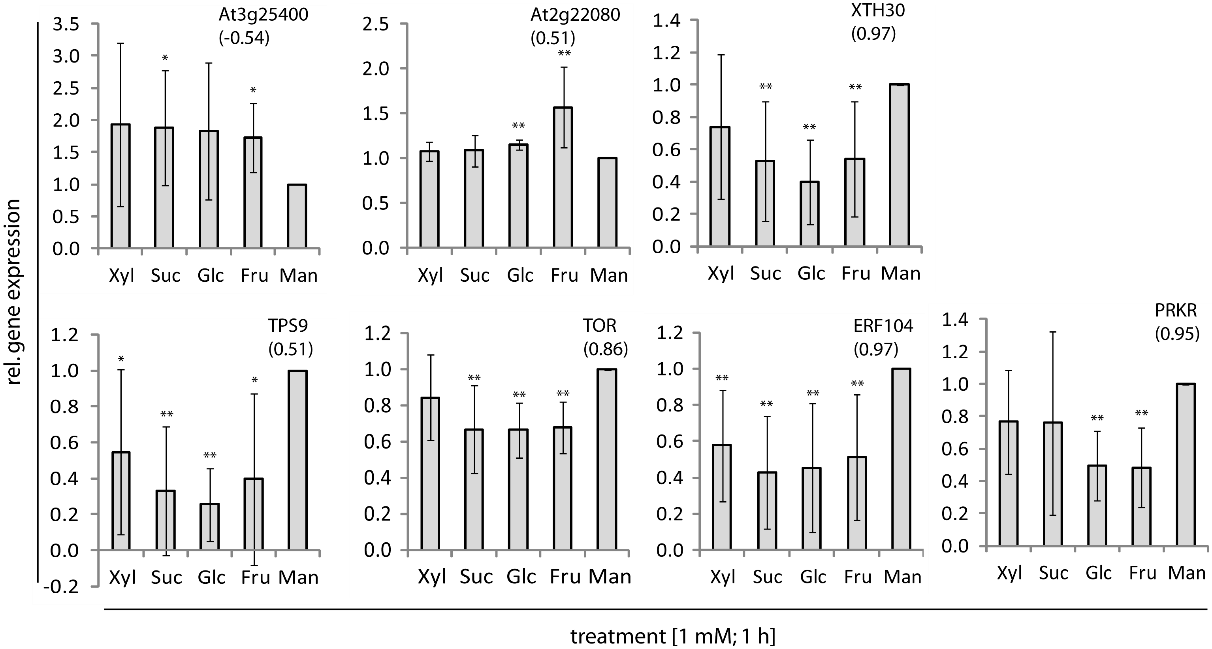


**Fig. S7**. Defined sets of sugars trigger significant changes in gene expression of selected sugar-responsive genes in 7-d-old *A.thaliana* cell culture. qPCR analysis of the expression of selected genes in response to Suc, Glc, Fru, Xyl and mannitol (Man) showed a sugar species-dependent regulation in response to Suc/Glc/Fru (*ERF104*, *XTH30*, *TPS9, TOR*), Glc/Fru (*At2g22080*, *PRKR*) and Suc/Fru (*At3g25400*). The numbers in parentheses indicate the correlation between the gene expression profile measured by the microarray and qPCR analysis. Significance: *t-*test; * α=0.05, ** α=0.01, n=8. See also **Fig.2B** and **Fig.3C** for examples of other genes.
