# Supplementary material for: Functional Dissection of Sugar Signals Affecting Gene Expression in Arabidopsis thaliana
Source: PLoS One. 2014 Jun 20;9(6):e100312. doi: 10.1371/journal.pone.0100312 (PMC4065033; doi:10.1371/journal.pone.0100312)
Supplement: Figure S8 — Effects of sugar analogues on expression of selected sugar-responsive genes in 7-d-old Xyl-grown A.thaliana cell culture. (DOCX) [file pone.0100312.s008.docx]

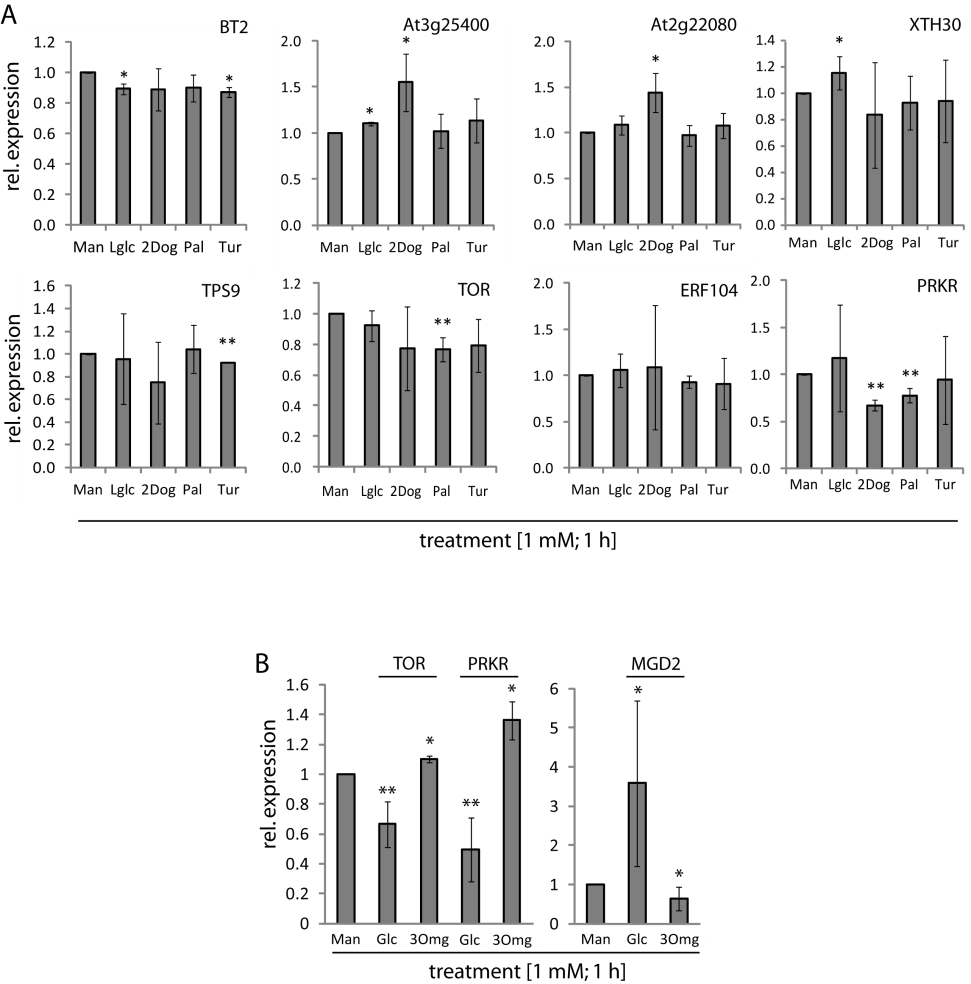


**Fig.S8.** Effects of sugar analogues on expression of selected sugar-responsive genes in 7-d-old Xyl-grown *A.thaliana* cell culture. **(A)** Effects of L-Glc, 2Dog, Pal and Tur (see also **Fig.4** for examples of other genes tested with those analogues). **(B)** Effects of 3OMG on expression of *TOR*, *PRKR* and *MGD2*. Man (mannitol), Lglc (L-Glc), 2Dog (2-deoxyglucose), Pal (palatinose), Tur (turanose), 3Omg (3-*O*-methylglucose). Significance: t-test; * α=0.05, ** α=0.01, n=5.
