# Supplementary material for: Functional Dissection of Sugar Signals Affecting Gene Expression in Arabidopsis thaliana
Source: PLoS One. 2014 Jun 20;9(6):e100312. doi: 10.1371/journal.pone.0100312 (PMC4065033; doi:10.1371/journal.pone.0100312)
Supplement: Figure S9 — The expression of cell-culture-selected sugar-responsive genes responds to light/dark conditions in intact plants. (DOCX) [file pone.0100312.s009.docx]

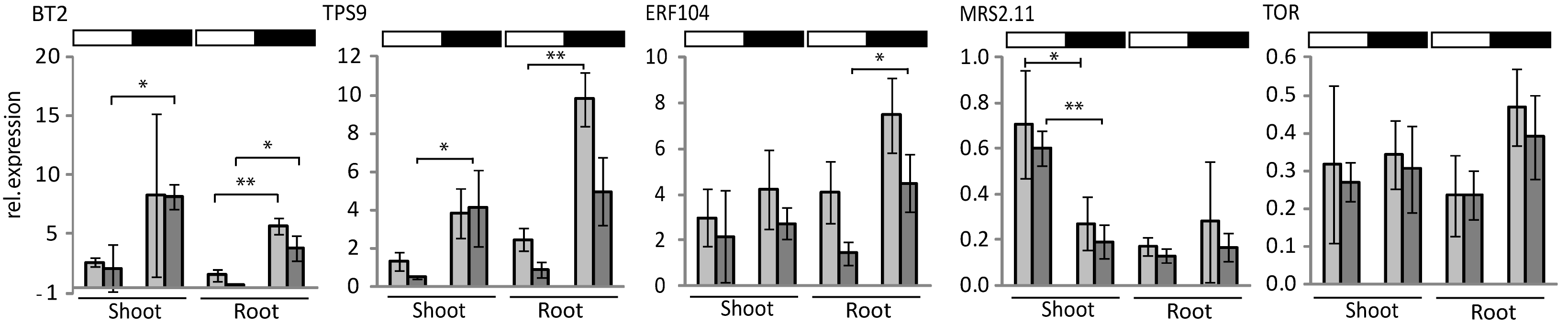


**Fig. S9.** The expression of cell-culture-selected sugar-responsive genes responds to light/ dark conditions in intact plants. Shoots and roots from 13-day-old *A. thaliana* seedlings of wt (light bar) and *pgm1* (dark bar), grown at 16/8 h photoperiod, were harvested at the stages of high (end of 16 h day, white box) and low (end of 14 h extended night, black box) carbon availability. Significance: *t-*test; *α=0.05, **α=0.01, n=3. See also **Fig.6B** for examples of other genes.
