# Supplementary figures and images for: Functional Dissection of Sugar Signals Affecting Gene Expression in Arabidopsis thaliana
Source: PLoS One. 2014 Jun 20;9(6):e100312. doi: 10.1371/journal.pone.0100312 (PMC4065033; doi:10.1371/journal.pone.0100312)

**Table S1:** Information on primer sequences used for qPCR analyses conducted within the study.


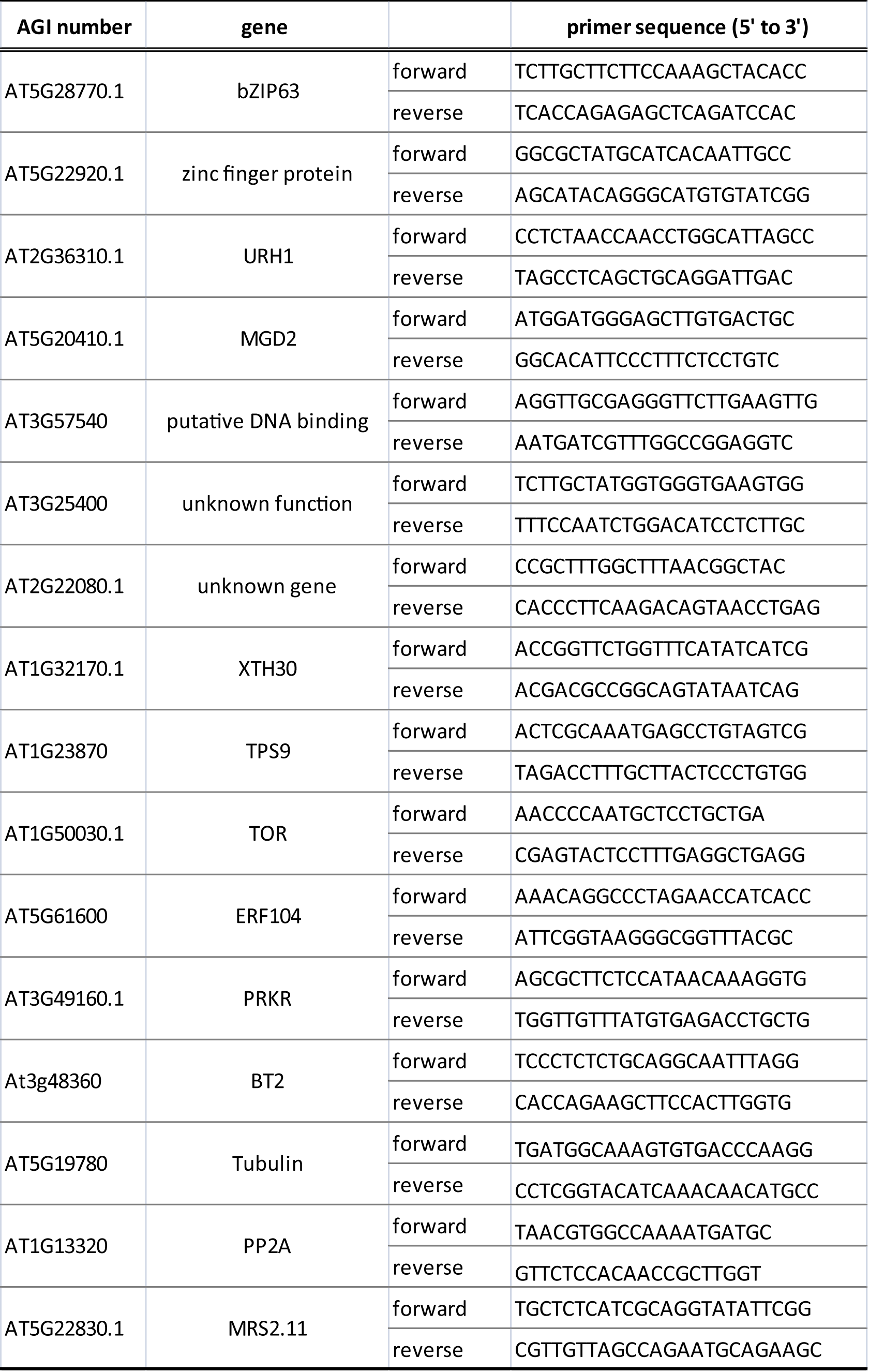

Supplement: Table S1 — Information on primer sequences used for qPCR analyses conducted within the study. (DOCX) [file pone.0100312.s010.docx]
