# Supplementary material for: Functional Dissection of Sugar Signals Affecting Gene Expression in Arabidopsis thaliana
Source: PLoS One. 2014 Jun 20;9(6):e100312. doi: 10.1371/journal.pone.0100312 (PMC4065033; doi:10.1371/journal.pone.0100312)
Supplement: Table S2 — Summary of sugar regulation of selected genes in A.thaliana cell culture. (DOCX) [file pone.0100312.s011.docx]

**Table S2**: Summary of sugar regulation of selected genes in *A.thaliana* cell culture. All sugars and sugar analogues were applied to the culture at 1 mM concentrations for 1 h.

**__________________________________________________________________________________**

**Effect of added sugar**

**Gene Fru D-Glc Suc Effective analogue Sugar-specific effect confirmed**

**_____________________________________________________________________________________**

*bZIP63*  ++ ++ ++ 2Dog Glc (HXK)

*At5g22920*  ++ ++ ++ 2Dog & Tur Glc (HXK) & Suc

*URH1* ++ ++ - none Unclear

*MGD2** ++ + + 2Dog Glc (HXK)

*At3g57540* ++ - - none Fru

*At3g25400* + - + L-Glc & 2Dog Unclear

*At2g22080* ++ ++ - 2Dog Glc (HXK)

*XTH30* ++ ++ ++ L-Glc Glc**

*TPS9* + ++ ++ Tur Suc

*TOR** ++ ++ ++ Pal Suc**

*ERF104* ++ ++ ++ none Unclear

*PRKR** ++ ++ - 2Dog & Pal Glc (HXK)

*BT2 ++ ++ ++* L-Glc & Tur Glc** & Suc

__________________________________________________________________________________

*Genes affected by 3OMG; its addition induced expression in the opposite direction than with sugars.

**Extracellular sensing

“Sugar-specific effects” were rationalized mostly from the effects of sugar analogues. Since we did not have Fru analogue, Fru effects could reflect **(i)** a Fru-specific pathway, responding either to Fru or some Fru-derived metabolite; or **(ii)** being an indirect result of some internal metabolism of Fru to Glc and Suc. In most cases, we could not distinguish between those possibilities. However, for *At3g57540* gene, the effect of Fru must reflect a Fru-specific pathway or a pathway specifically sensing a Fru-derived metabolite. For all genes studied, Suc effects, unless confirmed by Tur or Pal treatments, were likely indirect, resulting from limited Suc hydrolysis to Glc and Fru (see **Fig.S2**). For both *URH1* and *ERF104*, sensing of sugars likely occurs via a HXK-independent pathway, but other details are unknown. In the Table, we indicated (by a single star) genes whose expression was affected by 3OMG, but – given controversy as to whether 3OMG is sensed as sugar in plants (see the manuscript) - those results were not taken into account in the “Sugar-specific effect confirmed” panel. 2Dog, 2-deoxyglucose; 3OMG, 3-*O*-methylglucose; Pal, palatinose; Tur, turanose.
